# Impact of estrogen receptor gene polymorphisms and mRNA levels on obesity and lipolysis – a cohort study

**DOI:** 10.1186/1471-2350-8-73

**Published:** 2007-12-04

**Authors:** Maria Nilsson, Ingrid Dahlman, Hong Jiao, Jan-Åke Gustafsson, Peter Arner, Karin Dahlman-Wright

**Affiliations:** 1 Departments of Biosciences and Nutrition, Karolinska Institutet, S-141 57 Huddinge, Sweden; 2 Department of Medicine at Huddinge, Karolinska Institutet, S-141 86 Stockholm, Sweden

## Abstract

**Background:**

The estrogen receptors α and β (*ESR1, ESR2*) have been implicated in adiposity, lipid metabolism and feeding behaviour. In this report we analyse *ESR1 *and *ESR2 *gene single nucleotide polymorphisms (SNPs) for association with obesity. We also relate adipose tissue *ESR1 *mRNA levels and *ESR1 *SNPs to adipocyte lipolysis and lipogenesis phenotypes.

**Methods:**

23 *ESR1 *and 11 *ESR2 *tag-SNPs, covering most of the common haplotype variation in each gene according to HAPMAP data, were analysed by Chi^2 ^for association with obesity in a cohort comprising 705 adults with severe obesity and 402 lean individuals. Results were replicated in a cohort comprising 837 obese and 613 lean subjects. About 80% of both cohorts comprised women and 20% men. Adipose tissue *ESR1 *mRNA was quantified in 122 women and related to lipolysis and lipogenesis by multiple regression. *ESR1 *SNPs were analysed for association with adipocyte lipolysis and lipogenesis phenotypes in 204 obese women by simple regression.

**Results:**

No *ESR1 *SNP was associated with obesity. Five *ESR2 *SNPs displayed nominal significant allelic association with obesity in women and one in men. The two *ESR2 *SNPs associated with obesity with nominal P value < 0.01 were genotyped in a second cohort where no association with obesity was observed. There was an inverse correlation between *ESR1 *mRNA levels in abdominal subcutaneous (sc) adipose tissue and basal lipolysis, as well as responsiveness to adrenoceptor agonists independent of age and BMI (P value 0.009–0.045). *ESR1 *rs532010 was associated with lipolytic sensitivity to noradrenaline (nominal P value 0.012), and *ESR1 *rs1884051 with responsiveness to the non-selective beta-adrenoceptor agonist isoprenaline (nominal P value 0.05). These associations became non-significant after Bonferroni correction.

**Conclusion:**

*ESR1 *gene alleles are unlikely to be a major cause of obesity in women. A minor importance of *ESR2 *on severe obesity cannot be excluded. The inverse correlation between *ESR1 *mRNA levels and lipolytic responsiveness to adrenoceptor agonists implies that low adipose tissue *ESR1 *levels attenuate catecholamine resistance in sc fat cells of obese women hereby contributing to loss of sc and gain of visceral fat. There is no evidence for a genetic impact of *ESR1 *on lipolysis or lipogenesis.

## Background

The prevalence of obesity has reached epidemic proportions and is of great public health concern. Its metabolic complications, such as dyslipidemia and type 2 diabetes (T2D), represent a significant cost for the society. Though obesity is often triggered by life style, the importance of genetic predisposition has been clearly demonstrated [1]. Defining gene alleles that are associated with obesity will contribute to our understanding of the underlying mechanisms behind its development and potentially provide therapeutic targets.

Estrogen signalling is implicated in both central, that is regulation of food intake, and peripheral pathways protecting against adiposity. Low estrogen levels following menopause are associated with loss of subcutaneous (sc) and gain of visceral fat [2]. Estrogens signal via the transcription factors estrogen receptors α and β (ESR1, ESR2) [3]. Silencing of the *ESR1 *gene in mice renders an obese phenotype [4]. Both *ESR1 *and *ESR2 *are expressed in the hypothalamus, a brain area known to be involved in the regulation of appetite and satiety [5], whilst adipocytes express mainly *ESR1 *[6]. In the brain, *ESR2 *has been implicated in mediating the effects of estrogen on food intake [7]. As for peripheral effects of estrogens, hormone replacement therapy (HRT) has been shown to inhibit Epinephrine-stimulated lipolysis in human sc adipose tissue [8]. Estrogen, signalling through ESR1, lowers the lipolytic response in sc fat by increasing the number of antilipolytic α2-adrenoceptors [9].

Human genetic studies support a role for the *ESR *genes in regulation of body weight and certain aspects of the metabolic syndrome. Body mass index (BMI) (LOD 4.6, 4.2 and 2.8), waist circumference (LOD 3.3) and high-density lipoprotein (HDL) cholesterol levels have been linked to the chromosomal region harbouring *ESR1 *[10-13]. Among four investigated *ESR1 *polymorphisms, rs2234693 and rs9340799 were associated with waist circumference and rs1801132 with BMI in men, but not women [14]. In another study, *ESR1 *rs9340799, but not rs2234693, was associated with waist and BMI in Japanese women but not men [15]. Furthermore, neither rs2234693 nor rs9340799 was associated with obesity in Swedish women [16]. More recently, among 17 investigated *ESR1 *polymorphisms, rs6902771, rs2431260 and rs2175898 were associated with BMI in African Americans [17]. Among these same 17 *ESR1 *polymorphisms, rs1709183 and rs2431260 were associated with T2D in European Americans and rs1033182 in African Americans [18]. *ESR2 *has primarily been investigated in eating disorders. *ESR2 *rs928554 and rs4986938, but not rs1256049, have been associated with bulimic behavior [19] and rs1256049, but not rs4986938, with anorexia nervosa [20]. Rosenkrantz et al screened the coding region of *ESR2 *in probands of different weight extremes, but identified no mutations associated with the studied phenotypes [21].

We have recently reported that *ESR1 *mRNA expression levels in sc adipose tissue and isolated adipocytes from premenopausal women are inversely correlated with BMI [16]. However, whether *ESR1 *protects against obesity or is upregulated as a result of low BMI remains to be established. In this report we analyse polymorphisms covering most of the common haplotype variation in the *ESR1 *and *ESR2 *genes for association with obesity in two large cohorts of Swedish Caucasians. We also investigate if *ESR1 *is important for lipid turnover in human adiposity by analysing mRNA levels and gene polymorphisms for association with adipose tissue lipolysis and lipogenesis phenotypes in a third cohort of women., Table 1. To our knowledge, this represents the first extensive analysis of the impact of variations in the *ESR1 *and *ESR2 *genes for the development of obesity.

**Table 1:** Sample phenotypic distributions

Name Phenotype	Sample 1 Obesity		Sample 2 Obesity replication		Sample 3^a ^Fat cells
Obese	Women (N = 581)	Men (N = 124)	Women (N = 689)	Men (N = 148)	Women (N = 216)
Age (years)	41 ± 12	45 ± 13	43 ± 11	45 ± 12	38 ± 10
BMI (kg/m^2^)	43 ± 5	45 ± 5	37 ± 5	45 ± 5	38 ± 5
HOMA_IR_	5.3 ± 5.7	10.5 ± 16.5	4.5 ± 5.7	8.2 ± 5.5	3.8 ± 2.8
Pl-cholesterol (mmol/l)	5.2 ± 1.	5.2 ± 1.2	5.3 ± 1.1	5.2 ± 1.1	5.2 ± 1.1
Pl-HDL cholesterol (mmol/l)	1.2 ± 0.3	1.0 ± 0.2	1.3 ± 0.3	1.1 ± 0.3	1.2 ± 0.3
Pl-triglycerides (mmol/l)	1.7 ± 0.9	2.3 ± 2.3	1.7 ± 1.0	2.0 ± 1.2	1.6 ± 1.0
					
Lean	Women (N = 338)	Men (N = 64)	Women (N = 525)	Men (N = 88)	Women (N = 12)
Age (years)	50 ± 4	57 ± 8	39 ± 6	36 ± 11	33 ± 8
BMI (kg/m^2^)	22 ± 2	23 ± 2	22 ± 3	23 ± 1	22 ± 2
HOMA_IR_	1.3 ± 0.7	1.6 ± 0.8	1.3 ± 1.0	1.4 ± 0.7	1.4 ± 0.9
Pl-cholesterol (mmol/l)	5.4 ± 1.0	5.7 ± 1.1	4.7 ± 0.8	4.9 ± 1.1	4.8 ± 0.7
Pl-HDL cholesterol (mmol/l)	1.7 ± 0.4	1.4 ± 0.4	1.6 ± 0.4	1.3 ± 0.5	1.6 ± 0.4
Pl-triglycerides (mmol/l)	0.9 ± 0.5	1.5 ± 1.0	0.9 ± 0.5	1.3 ± 1.4	0.8 ± 0.2

Values are mean ± SD

a) 99 subjects overlap between sample 1 and 3.

## Methods

### Subjects and clinical evaluation

Samples 1 and 2 were recruited for the purpose of studying genes underlying susceptibility to obesity, Table 1. In sample 1, the lean subjects were subjects > 45 years old and had never reached BMI > 25 kg/m^2 ^whereas the obese subjects had BMI > 30 kg/m^2 ^at < 20 years of age or morbid obesity, *i.e. *BMI > 40 kg/m^2^. These recruitment criteria resulted in more young obese than lean subjects, Table 1. The aim of selecting subjects with an extreme BMI phenotype in sample 1 was to enrich for a genetic impact on obesity [22]. This was also the purpose of recruiting young obese adults, since early onset of this disorder is believed to have a stronger genetic component due to reduced time of environmental impact [22]. The size of sample 1 provided 90% power to detect an allele with a frequency of 20% among controls and odds ratio (OR) ≥ 1.5 to develop obesity assuming a threshold P value of 0.05 and dominant impact on obesity [23]. 59 subjects in sample 1 were diagnosed with T2D according to self report, but otherwise the subjects were healthy and free of medication according to self-report. 381 subjects in sampe 1 were also included in our previously reported analysis of two *ESR1 *SNPs in obesity [16].

Sample 2 had less stringent inclusion criteria for obesity and leanness in comparison with sample 1. Sample 2 comprised healthy non-obese subjects > 25 years old with BMI < 25 kg/m^2 ^and obese individuals with BMI > 30 kg/m^2^, Table 1. 197 of the obese subjects in sample 2 had hypertension, 116 T2D, and 50 dyslipidemia according to self report. Differences in recruitment criteria resulted in phenotypic differences between sample 1 and 2, Table 1. The ratios between men and women were similar among obese and lean in both samples (17–21%).

Sample 3 comprised obese and lean otherwise healthy women recruited with the purpose of studying fat cell function, Table 1. Lipolysis and lipogenesis were investigated in 204 obese women in sample 3. 204 subjects provide approximately 75% power to detect an allele with a frequency of 20% and R^2 ^0.04 assuming a threshold P value of 0.05 and dominant impact on adipocyte phenotypes [23]. *ESR1 *mRNA levels in adipose tissue were quantified in the 122 women of sample 3 for which RNA was available. 99 women in sample 3 who met the criteria for obesity in sample 1 were included in both samples.

All subjects above were Caucasians and at least second generation Swedish. Obese subjects were recruited from an outpatient center for treatment of obesity or through local advertisement. All lean subjects were recruited through local advertisement. All subjects came to the laboratory in the morning after an overnight fast. A venous blood sample was obtained for extraction of genomic DNA and determination of serum insulin, as well as plasma glucose, triglycerides, total cholesterol, and HDL cholesterol as described [24,25]. Insulin resistance index HOMA_IR _(homeostasis model assessment) was calculated as fasting serum insulin (μU/ml) × fasting plasma glucose (mmol/l)/22.5 [26]. The ethical committee of the Karolinska University Hospital (Southern Campus) approved the study. It was explained in detail to each participant and her/his consent was obtained.

### Lipolysis and lipogenesis investigation

In the morning after an overnight fast a sc fat biopsy was obtained from the abdominal area by needle biopsy [27]. One part of the adipose tissue pieces was immediately frozen in liquid nitrogen for subsequent RNA analysis. Another piece was digested with collagenase to isolate fat cells and mean fat cell weight was determined [28]. Lipolysis in isolated fat cells was investigated as described [29,30]. Briefly, cell suspensions were incubated in the absence or presence of increasing concentrations of noradrenaline, the non-selective β-adrenoceptor agonist isoprenaline, or the α2-adrenoceptor selective agonist clonidine. At the end of incubation an aliquot of the medium was removed for analysis of glycerol release, which was used as an index of lipolysis.

Lipogenesis was investigated by determining the uptake of radio labeled glucose into lipids as described [31]. Briefly, diluted suspensions of fat cells were incubated in the absence or presence of increasing concentrations of crystalline human insulin (10^-15^–10^-6 ^mol/l) for 2 h at 37°C. Incubation was terminated by addition of sulphuric acid and radioactivity incorporated into lipids determined.

The concentration (log mol/l) of hormone or agonist causing half maximum effect was determined using logarithmic conversion of each concentration response curve. This value was converted to its negative form (pD_2_), which reflects hormone sensitivity. The maximum effect or responsiveness of hormone or agonist was determined as glycerol release or glucose incorporation into lipids at the maximum effective hormone concentration.

### mRNA quantification

Total RNA was prepared from adipose tissue and reverse transcribed as described [32]. All qPCR assays were run in triplicates on an ABI 7500 machine (Applied Biosystems, Foster City, CA, USA). A direct comparative method was used for data analysis with *GAPDH *as control gene (User Bulletin #2, Applied Biosystems). The amplification was repeated twice to confirm results. *ESR1 *was amplified by Taqman whereas a SYBRGreen assay was applied for *GAPDH*. Primer/probe sequences were as follows: *ESR1 *(F: 5'-AATATGCCCTTTTGCGATG-3'; R: 5'-ACAAAGCAAAGCTGCGACAA-3'; and Taqman probe: 5'-CTATTACTGATGTGACTCGGT-3'), GAPDH (F: 5'-TGACAACTTTGGTATCGTGGAAGG-3'; R: 5'-AGGCAGGGATGATGTTCTGGAGAG-3').

### SNP selection and genotyping

Genotype information for *ESR1 *and *ESR2 *and regions approximately 10 000 base pairs up- and downstream of these genes were downloaded from HAPMAP in May 2005 [33]. Genotype data for the population of individuals of European ancestry were visualized using Haploview [34]. We initially selected tag-SNPs defining all haplotypes with frequency > 5% from the block-by-block tags displayed in the HAPLOVIEW "Haplotypes" window. However, sometimes these tag-SNPs were exchanged to other SNPs on the same haplotype due to difficulties in designing genotyping assays. In addition, in regions not covered by common haplotypes we aimed to select one common SNP (allele frequency > 5%) every 5 000 base pair. No non-synonymous SNPs full-filling these criteria were detected in the *ESR *genes. In selecting between different SNPs we prioritized (1) Golden-gate validated assays, and (2) SNPs with high score according to Illumina, which indicate that the designed Illumina genotyping assays are highly likely to work [35]. Besides the HAPMAP SNPs we genotyped the *ESR2 *SNPs rs928554 and rs4986938, which previously have been associated with feeding behavior [19].

Samples 1 and 3 were genotyped using the Illumina technology at the SNP technology platform in Uppsala [36,35] except SNPs rs928554 and rs4986938 that were genotyped by RFLP as described [19]. Sample 2 was genotyped using matrix-assisted laser desorption/ionization time-of-flight (MALDI-TOF) mass spectrometry (SEQUENOM Inc., San Diego, California) as described [37]. Primers can be provided on request. The genotype call rate for all genotyping platforms was ≥ 97% and the accuracy was 99.99% according to duplicate analysis of, on average, 2% of the total genotypes. Hardy-Weinberg equilibrium (HWE) calculations were performed to ensure that each marker was within population equilibrium.

### Statistical analyses

Association between *ESR1 *mRNA levels and lipolysis and lipogenesis measures was assessed by multiple regression with age and BMI as additional independent variables. The Finetti software [38] using Pearson's goodness-of-fit Chi^2 ^was employed to test for allelic association between single SNPs and obesity. ANCOVA with age and BMI as covariates were used to analyze differences in insulin resistance and blood lipid phenotypes between genotypes. HOMA_IR_, Pl-HDL cholesterol, and Pl-triglycerides were ln-transformed before analysis to become normally distributed. For genetic analysis of adipocyte lipolysis and lipogenesis, logarithm transformed phenotypes were used as independent quantitative variables in linear regression models. Genotypes were indicated as character (nominal) with three levels, two different homozygous and heterozygous. There was no association between the adipocyte phenotypes and age or BMI in our sample and these variables were therefore not included in the regression model. Haplotypes were estimated and analyzed with Haploview [34]. LD was calculated as D'. We used HAPMAP Caucasian data to define haploblock limits. Association between haplotypes and obesity status was evaluated by Chi^2^. Haplotypes with frequency < 5% and individuals with > 50% missing genotypes were excluded in the analysis. We performed 10 000 permutations in Haploview to adjust P values to multiple tests.

## Results

### ESR genotyping results

**Figure 1:**
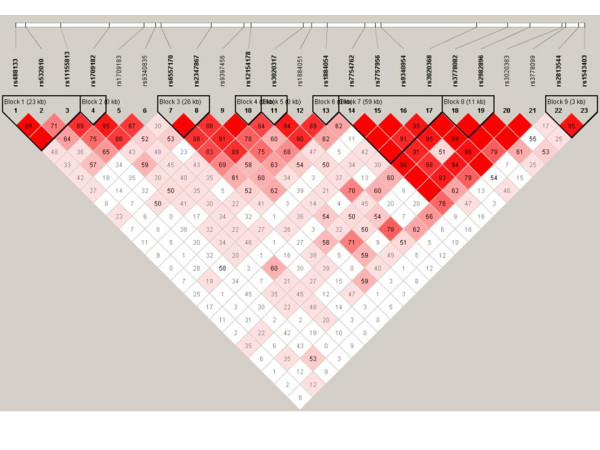
LD (D') between *ESR1 *SNP and haploblocks according to Caucasian HAPMAP data.

**Figure 2:**
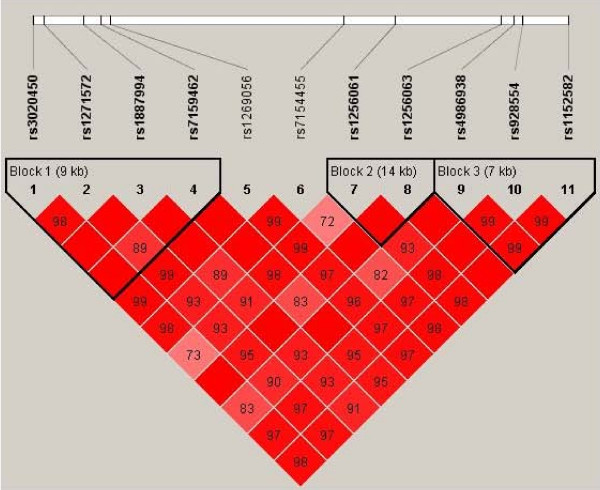
LD (D') between *ESR2 *SNP and haploblocks according to Caucasian HAPMAP data.

To investigate if *ESR1 *and *ESR2 *alleles contribute to susceptibility to obesity, polymorphisms covering the common variation in these genes were genotyped in sample 1, Table 2. For *ESR1*, 6 out of 29 (21%) Illumina genotyping assays failed, which is higher than the expected 10% failure rate. The majority represented Golden-gate validated assays indicating, according to the supplier, that the failure is due to interference between different SNP assays under the employed multiplex conditions. LD between *ESR *gene SNPs is shown in Figure 1 and 2, in which haploblock limits according to Caucasian HAPMAP data have been labeled. Genotyped *ESR1 *SNPs built 25 haplotypes, Table 3. These SNPs unambiguously identified 67% (20/30) of the common (> 5%) haplotypes in the *ESR1 *region in HAPMAP, representing 75% of the common haplotype variation in *ESR1 *according to HAPMAP data. Remaining ten *ESR1 *haplotypes in HAPMAP could in our sample not be separated from another haplotype, that is they were merged into five haplotypes. For *ESR2*, all genotyped assays were called successfully although one SNP, rs7154455, displayed a low genotyping call rate, 83%, Table 2. Thus there was 100% coverage of the common Caucasian *ESR2 *haplotypes in HAPMAP, Figure 2. All SNPs were in HWE.

**Table 2:** Genotyped *ESR1 *and *ESR2 *SNPs

Marker	Gene position	Chromosome position	Alleles	Common allele (%)	Call rate (%)	Chi^2 ^of HWE
*ESR1 *(Chromosome 6)						
rs488133	5'	152217558	C > T	68	98	0.45
rs532010	5'	152223032	A > G	65	98	0.95
rs11155813	intron 1	152241549	T > C	88	96	0.06
rs1709182	intron 2	152267471	T > C	66	96	0.81
rs1709183	intron 2	152286110	T > C	72	98	0.4
rs9340835	intron 2	152292045	G > A	68	98	0.06
rs6557170	intron 3	152295218	G > A	79	95	0.97
rs2347867	intron 3	152321964	A > G	68	98	0.17
rs9397456	intron 3	152339266	G > A	80	98	0.15
rs12154178	intron 3	152343194	A > C	72	98	0.59
rs3020317	intron 4	152370855	T > C	82	98	0.03
rs1884051	intron 4	152375393	A > G	71	98	0
rs1884054	intron 4	152383680	A > C	67	98	0.47
rs7754762	intron 4	152403651	T > A	87	97	1.61
rs7757956	intron 4	152409254	T > A	86	98	2.69
rs9340954	intron 4	152412286	T > G	70	98	2.89
rs3020368	intron 5	152463304	C > T	91	97	0.05
rs3778082	intron 6	152479778	G > A	86	95	1.76
rs2982896	intron 6	152491607	C > T	77	97	0.01
rs3020383	intron 7	152508893	G > C	92	97	0.28
rs3778099	intron 7	152510689	T > C	87	97	1.18
rs2813544	3'	152517696	A > G	79	98	0.88
rs1543403	3'	152520818	G > C	52	96	1.48
*ESR2 *(Chromosome 14)						
rs3020450	5'	63838055	C > T	70	93	6.29^a^
rs1271572	5'	63831670	C > A	54	98	1.03
rs1887994	intron 1	63830364	C > A	89	97	0.28
rs7159462	intron 1	63828629	C > T	92	97	0.14
rs1269056	intron 3	63813643	C > T	56	97	1.3
rs7154455	intron 3	63806413	G > C	64	83	0.23
rs1256061	intron 7	63773346	G > T	50	97	1.89
rs1256063	intron 7	63771970	G > A	91	94	0.27
rs4986938	3'	63769569	G > A	62	100	0.56
rs928554	3'	63763948	A > G	56	100	2.8
rs1152582	3'	63762383	C > G	56	98	0.71

a) HWE among controls n.s.

**Table 3:** Association of *ESR1 *and *ESR2 *haplotypes with obesity

		Cases		Controls		
Haploblock	%	Specific haplotype (n)	Other (n)	Specific haplotype (n)	Other (n)	P value
*ESR1*						
rs488133-rs532010-rs11155813						
CGT	34	449	917	288	508	0.12
TAT	33	455	911	250	546	0.37
CAT	21	285	1081	167	629	0.96
CAC	11	158	1208	82	714	0.36
rs1709182						
T	67	925	441	516	282	0.14
C	33	441	925	282	516	0.14
rs6557170-rs2347867						
GA	68	916	450	549	249	0.40
AG	21	299	1067	165	633	0.52
GG	11	151	1215	84	714	0.68
rs12154178						
A	18	976	390	575	223	0.76
C	28	390	976	223	575	0.76
rs3020317						
T	82	1116	250	652	146	1.00
C	18	250	1116	146	652	1.00
rs1884054						
A	67	920	446	531	267	0.70
C	33	446	920	267	531	0.70
rs7754762-rs7757956-rs9340954-rs3020368						
TTTC	69	947	420	557	239	0.75
ATGC	13	185	1181	99	697	0.47
TAGT	8	113	1253	69	727	0.77
TAGC	5	67	1299	43	753	0.59
rs3778082-rs2982896						
GC	63	862	502	502	296	0.90
GT	23	310	1054	197	601	0.30
AC	14	192	1172	99	699	0.27
rs2813544-rs1543403						
AC	48	640	727	396	402	0.21
AG	31	419	948	248	550	0.82
GG	21	301	1065	152	646	0.10
*ESR2*						
rs3020450-rs1271572-rs1887994-rs7159462						
CACC	46	639	727	346	452	0.12
TCCC	29	372	994	254	544	0.024*
CCAC	11	153	1213	79	719	0.33
CCCT	8	116	1251	65	733	0.83
CCCC	6	81	1285	49	749	0.86
rs1256061-rs1256063						
TG	50	671	689	401	397	0.68
GG	41	547	814	331	467	0.57
GA	10	142	1218	66	732	0.10
rs4986938-rs928554-rs1152582						
GGG	44	619	747	333	465	0.10
AAC	38	502	864	310	488	0.32
GAC	18	233	1133	149	649	0.35

### Analysis of ESR SNPs and haplotypes in obesity

No *ESR1 *SNP displayed significant allelic association with obesity, but one SNP, rs2813544, was associated with obesity in analysis of homozygous subjects only, nominal P value 0.05, Table 4. Two *ESR2 *SNPs, rs7154455 and rs3020450, displayed allelic association with obesity in the joint analysis of women and men, nominal P value 0.0003–0.01, Table 4. In permutation test, the association between rs7154455 and obesity remained significant (P value 0.0022), whereas the result for rs3020450 became borderline significant (P value 0.064). Both SNPs were associated with obesity in women, but not in men. For *ESR2 *SNPs rs1152582, rs1271572 and rs1269056, allelic associations with obesity were observed in women, nominal P value 0.03–0.05, but not in joint analysis of women and men, Table 4. *ESR2 *SNP rs4986938 was associated with obesity in men only, nominal P value 0.05. rs7154455 and rs3020450 were genotyped in sample 2 where no association with obesity was observed, Table 4. Nor was there any association with obesity in analysis of pooled samples 1 and 2 (results not shown). No *ESR *SNP was associated with HOMA_IR_, Pl-cholesterol, Pl-HDL cholesterol, and Pl-triglycerides with P < 0.0125, and did thus not remain significant after Bonferroni correction for analysis of several SNPs (results not shown).

**Table 4:** Association between *ESR *SNPs and obesity in samples 1 and 2^a^

Gene	SNP	Controls (n)			Cases (n)			Allele Frequency		Heterozygous		Homozygous		Recessive model		Armitage's trend test	
		22^b^	21	11	22	21	11	[2]<-> [1]^c^	P value	[22]<-> [12]	P value	[22]<-> [11]	P value	[11+12]<-> [22]	P value	OR	P value
Sample 1																	
*ESR1*	rs2813544	10	134	255	33	242	408	0.82 [0.66–1.02]	0,08	0.55 [0.26–1.14]	0,11	0.48 [0.24–1.00]	**0,05**	0.51 [0.25–1.04]	0,06	0,79	0,07
	Women	8	114	213	29	194	339	0.83 [0.66–1.06]	0,13	0.47 [0.21–1.06]	0,06	0.44 [0.20–0.98]	**0,04**	0.45 [0.20–1.00]	**0,04**	0,79	0,13
	Men	2	20	42	4	48	69	0.77 [0.45–1.31]	0,33	1.20 [0.20–7.08]	0,84	0.82 [0.14–4.68]	0,82	0.94 [0.17–5.30]	0,95	0,78	0,31
*ESR2*	rs7154455	107	167	47	273	260	62	0.70 [0.57–0.85]	**0,0003**	0.61 [0.45–0.82]	**0,001**	0.52 [0.33–0.80]	**0,003**	0.59 [0.44–0.78]	**0,0002**	0,7	**0,0003**
	Women	84	144	43	223	219	45	0.63 [0.51–0.78]	**0,00003**	0.57 [0.41–0.80]	**0,0008**	0.39 [0.24–0.64]	**0,0001**	0.53 [0.39–0.73]	**0,00007**	0,62	**0,00002**
	Men	23	23	4	50	41	17	1.18 [0.71–1.97]	0,51	0.82 [0.40–1.67]	0,58	2.0 [0.59–6.46]	0,27	0.99 [0.50–1.94]	0,97	1,26	0,53
	rs3020450	177	163	44	353	237	59	0.78 [0.64–0.94]	**0,01**	0.73 [0.56–0.95]	**0,02**	0.67 [0.44–1.03]	0,07	0.72 [0.56–0.92]	**0,01**	0,8	**0,01**
	Women	147	139	41	288	202	42	0.72 [0.58–0.89]	**0,002**	0.74 [0.55–1.00]	**0,05**	0.52 [0.33–0.84]	**0,007**	0.69 [0.52–0.91]	**0,009**	0,73	**0,003**
	Men	30	24	3	65	35	17	1.17 [0.71–1.94]	0,54	0.67 [0.34–1.32]	0,25	2.61 [0.71–9.61]	0,14	0.89 [0.47–1.68]	0,72	1,32	0,57
*ESR2*	Women																
	rs1152582	54	171	110	127	274	161	0.80 [0.66–0.98]	**0,03**	0.68 [0.47–1.00]	**0,04**	0.62 [0.42–0.93]	**0,01**	0.66 [0.46–0.94]	**0,02**	0,8	**0,03**
	rs1271572	59	172	104	130	281	151	0.82 [0.68–1.00]	**0,05**	0.74 [0.52–1.06]	0,1	0.66 [0.44–1.00]	**0,04**	0.71 [0.50–1.00]	**0,05**	0,82	**0,04**
	rs1269056	53	175	107	127	275	158	0.81 [0.66–0.98]	**0,03**	0.66 [0.45–0.95]	**0,03**	0.62 [0.41–0.92]	**0,02**	0.64 [0.45–0.91]	**0,01**	0,8	**0,03**
*ESR2*	Men																
	rs4986938	4	30	30	20	59	45	0.64 [0.40–1.00]	**0,05**	0.39 [0.12–1.26]	0,11	0.30 [0.09–0.96]	**0,04**	0.35 [0.11–1.06]	**0,05**	0,6	**0,05**
Sample 2																	
*ESR2*	rs3020450	326	245	41	410	332	60	1.08 [0.91–1.27]	0,39	1.08 [0.86–1.34]	0,51	1.16 [0.76–1.78]	0,48	1.09 [0.88–1.34]	0,42	1.08	0,38
	Women	273	216	35	346	282	53	1.06 [0.89–1.27]	0,42	1.03 [0.81–1.31]	0,81	1.20 [0.76–1.88]	0,44	1.05 [0.84–1.32]	0,65	1.07	0,51
	Men	53	29	6	64	50	7	1.18 [0.75–1.86]	0,46	1.42 [0.80–2.56]	0,23	0.97 [0.31–3.05]	0,95	1.35 [0.77–2.35]	0,29	1.11	0,46
	rs7154455	318	248	46	399	341	63	1.06 [0.90–1.26]	0,45	1.10 [0.88–1.37]	0,42	1.09 [0.73–1.64]	0,67	1.09 [0.89–1.35]	0,40	1.06	0,44
	Women	268	216	40	337	287	57	1.06 [0.89–1.27]	0,52	1.06 [0.83–1.34]	0,65	1.13 [0.73–1.75]	0,57	1.07 [0.85–1.34]	0,57	1.06	0,52
	Men	50	32	6	62	54	6	1.11 [0.71–1.73]	0,64	1.36 [0.77–2.42]	0,29	0.81 [0.24–2.65]	0,72	1.27 [0.73–2.21]	0,74	1.05	0,63

a) Shown are SNPs with P value < 0.05 in at least one analysis.

b) 1 and 2 represent the two alleles of each SNP.

c) OR (95% confidence interval)

In sample 1, no *ESR1 *haplotype was associated with obesity, Table 3. One common *ESR2 *haplotype, TCCC (frequency 29%) at SNPs rs3020450-rs1271572-rs1887994-rs7159462 was associated with obesity, nominal P value 0.024, Table 3. TCCC was captured by SNP rs3020450. rs3020450 was genotyped in sample 2, where we were unable to confirm the association observed in sample 1.

### Relation of ESR1 SNPs and mRNA levels to adipocyte lipolysis and lipogenesis phenotypes

**Figure 3:**
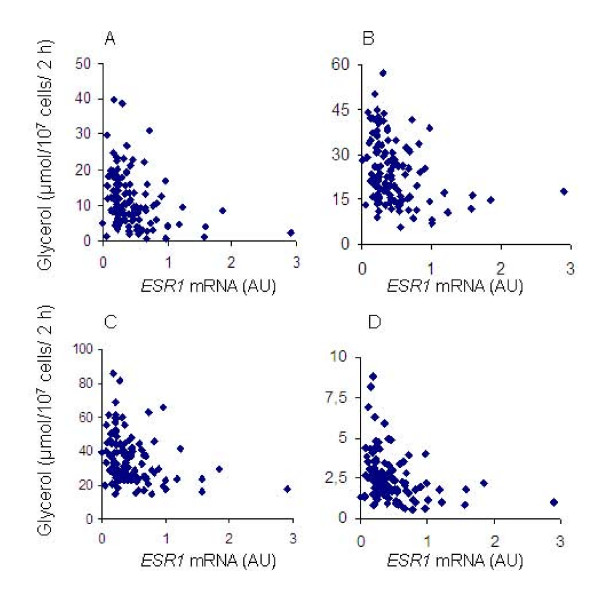
*ESR1 *mRNA levels in abdominal sc adipose tissue plotted against (A) basal lipolysis, response to (B) noradrenaline, (C) the non-selective β-adrenoceptor agonist isoprenaline, and (D) the α2-adrenoceptor selective agonist clonidine.

In 122 women with a large variation in BMI, part of sample 3, there was an inverse correlation between *ESR1 *mRNA levels in abdominal sc adipose tissue and adipocyte basal lipolysis as well as responsiveness to noradrenaline and more selective adrenoceptor agonists, Figure 3. In multiple regression, including BMI, age, and *ESR1 *mRNA levels as independent variables, *ESR1 *levels were an independent factor regulating basal lipolysis (P = 0.018), as well as responsiveness to noradrenaline (P = 0.013), Table 5. Association between *ESR1 *mRNA levels and lipolytic responsiveness was stronger for the α2-adrenoceptor selective agonist clonidine (P = 0.009), than for the non-selective β-adrenoceptor agonist isoprenaline (P = 0.045), Table 5. There was no association between *ESR1 *mRNA levels and sensitivity of lipolysis to adrenoceptor agonists, nor between *ESR1 *mRNA levels and measures of lipogenesis. We next analysed *ESR1 *SNPs for impact on adipocyte lipolysis and lipogenesis in the 204 obese women of sample 3, for which these phenotypes had been investigated. No SNP was associated with basal lipolysis (results not shown). One *ESR1 *SNP, rs532010, was associated with lipolytic sensitivity to noradrenaline, nominal P value 0.012, Table 6. Another *ESR1 *SNP, rs1884051, was associated with responsiveness to the non-selective beta-adrenoceptor agonist isoprenaline, nominal P value 0.05. These SNP associations became non-significant after Bonferroni correction for analysis of several SNPs. No *ESR1 *SNP was associated with lipogenesis (results not shown).

**Table 5:** Regression of basal lipolysis and responsiveness to adrenoceptor agonist on *ERS1 *mRNA levels

	Coefficient ± SE	Std. Coeff.	T-value	P value
Basal lipolysis				
Intercept	2.34 ± 4.54	2.34	0.51	0.61
*ESR1 *(AU)	-3.90 ± 1.62	-0.20	-2.40	0.018
BMI (kg/m^2^)	0.44 ± 0.10	0.39	4.61	<0.0001
Age (years)	-0.14 ± 0.08	-0.15	-1.86	.065
Response to noradrenaline				
Intercept	13.84 ± 6.40	13.84	2.16	.033
*ESR1 *(AU)	-5.72 ± 2.26	-0.21	-2.53	.013
BMI (kg/m^2^)	0.58 ± 0.13	0.37	4.38	<0.0001
Age (years)	-0.22 ± 0.11	-.174	-2.12	.036
Response to isoprenaline				
Intercept	15.44 ± 8.72	15.44	1.77	0.079
*ESR1 *(AU)	-6.31 ± 3.11	-0.18	-2.03	0.045
BMI (kg/m^2^)	0.78 ± 0.18	0.37	4.28	<.0001
Age (years)	-0.16 ± 0.14	-0.10	-1.14	0.26
Response to clonidine				
Intercept	0.99 ± 0.92	0.99	1.08	0.28
*ESR1 *(AU)	-0.87 ± 0.33	-0.23	-2.65	0.0091
BMI (kg/m^2^)	0.07 ± 0.02	0.31	3.57	0.0005
Age (years)	-0.02 ± 0.02	-0.08	-1.00	0.32

**Table 6:** Association between *ESR1 *SNPs and lipolysis and lipogenesis phenotypes^a^

	Source	Sum of Squares	F Ratio	Prob > F
Noradrenaline pD2, lipolysis ^b^				
*ESR1*	rs532010	0.045	4.51	0.012
Response to Isoprenaline, lipolysis ^c^				
*ESR1*	rs1884051	0.219	3.02	0.050

a) For quantitative analysis of lipolysis and lipogenesis, logarithm transformed phenotypes were used as independent quantitative variables in linear regression models. Genotypes were indicated as character (nominal) with three levels, two alternative homozygous and heterozygous.

b) The concentration (log mol/l) of hormone or agonist causing half maximum effect was determined using logistic conversion of each concentration response curve. This value was converted to its negative form (pD_2_), which reflects hormone sensitivity.

c) Lipolysis is expressed as mmol of glycerol/2 h/10^7 ^cells.

## Discussion

Estrogen signalling has been implicated in regulation of human adiposity [4]. There is evidence that *ESR1 *acts in fat cells, whereas *ESR2 *mediates the effects of estrogen on food intake [7,9]. We here report that *ESR1 *mRNA levels in adipose tissue were inversely correlated with basal lipolysis and adrenoceptor responsiveness in obese women. In sample 1, two *ESR2 *SNPs were associated with obesity with nominal P value < 0.01. However, these associations were not confirmed in sample 2. No *ESR1 *SNP displayed allelic association with obesity or lipolysis.

Although no *ESR1 *SNP displayed allelic association with obesity, we cannot completely exclude an impact of *ESR1 *alleles on female obesity since genotyped SNPs failed to capture a few common haplotypes in the region of the *ESR1*gene.

Two *ESR2 *SNPs, rs7154455 and rs3020450 were associated with obesity with nominal P value < 0.01. The call rate for rs7154455 was only 83% adding some uncertainty to this association. rs7154455 and rs3020450 were genotyped in sample 2. In sample 2, as well as in pooled analysis of sample 1 and 2, no association with obesity was observed. This suggests that the allelic association of the two *ESR2 *SNPs in sample 1 was spurious. However, since recruitment criteria for sample 2 were based on less stringent definitions of obesity and leanness, and included subjects with metabolic complications of obesity, we cannot exclude that the *ESR2 *SNPs have a modest impact on more extreme forms of uncomplicated obesity such as in sample 1. In addition, this study was not designed to study potential gene-environment interactions. This is particularly important for ESR2, which has been implicated in mediating the effects of estrogen on food intake. Furthermore, our cohort of men was too small to exclude a male-specific impact of *ESR1 *and *ESR2 *on obesity. This might explain the difference between our results and the association of *ESR1 *and male obesity previously reported [14].

We have reported before that *ESR1 *rs2234693 and rs9340799 are not associated with obesity in a cohort of Swedish women partially overlapping with the cohort studied in this project [16]. We therefore did not genotype rs2234693 and rs9340799 in this study. *ESR1 *rs1801132, reported to be associated with BMI in men in the Framingham Heart Study [14], is according to the Caucasian HAPMAP data on the same haplotype as rs9397456, which was genotyped in this project. We did not genotype the three *ESR1 *SNP reported to be associated with BMI in African Americans [17]; however the reported BMI associated SNP rs6902771 is according to HAPMAP on the same haplotype as rs2234693, which we genotyped. We genotyped rs1709183, but not rs2431260, reported to be associated with T2D [18]. Finally, we genotyped rs928554 and rs4986938, but not rs1256049, implicated in eating disorders [19,20]. SNPs genotyped in our present project, that previously have been associated with obesity related and eating disorders, were not associated with obesity in the present project.

Human obesity is associated with increased rate of basal lipolysis in all fat depots and lipolytic resistance to catecholamines in sc fat [39]. Our finding that *ESR1 *mRNA levels in adipose tissue are inversely correlated with rate of basal lipolysis might suggest that low adipose tissue *ESR1 *levels, as observed in obesity [16], increases basal lipolysis. In addition, the inverse correlation between *ESR1 *mRNA levels and lipolytic responsiveness to catecholamines implies that low adipose tissue *ESR1 *levels among obese counteracts catecholamine resistance in sc fat cells of obese women. Catecholamine resistance among obese is specific to sc fat [39]. Attenuation of lipolytic catecholamine resistance may explain why low estrogen signalling is associated with fat redistribution with loss of sc and gain of visceral fat [2]. In our analyses, there was no evidence for a genetic impact of *ESR1 *on lipolysis or lipogenesis.

## Conclusion

Common *ESR1 *gene alleles are unlikely to contribute to obesity in women, whereas a minor importance of *ESR2 *on severe obesity cannot be excluded. The male subset in our sample was too small to exclude that *ESRs *have an impact on obesity in men. Low *ESR1 *mRNA levels in adipose tissue may counteract catecholamine resistance in fat cells of obese women hereby contributing to loss of sc and gain of visceral fat [39]. In our analysis of obese women there was no evidence for a genetic impact of *ESR1 *on lipolysis or lipogenesis. In future research, it will be important to investigate if there are interactions between ESR2 gene alleles and environmental factors such as food intake.

## Competing interests

The author(s) declare that they have no competing interests, except in case of Jan-Åke Gustafsson who is shareholder, research grant receiver and consultant of KaroBio AB.

## Authors' contributions

MN performed the mRNA measurements and RFLP, analysed data, and drafted the manuscript. ID designed the genetic study, analysed data, and drafted the manuscript together with MN. HJ analysed data. JAG helped to draft the manuscript. PA collected the obese and lean samples and was responsible for the fat cell studies. KDW designed the study and helped to draft the manuscript. All authors read and approved the final manuscript.

## Pre-publication history

The pre-publication history for this paper can be accessed here:


